# Bee-Virus for Acute Rheumatism

**Published:** 1894-02

**Authors:** James Wood

**Affiliations:** Brooklyn, N. Y.


### BEE-VIRUS FOR ACUTE RHEUMATISM.

*To the Editor of The Medical News.*

Sir:—The following extract appears in the *Bulletin* of the Division of Entomology of the Department of Agriculture (vol. V, No. 5):

"Mr. John Worthington, United States Consul at Malta, has sent us a clipping from the *Malta Standard*, of April 11th, which states that the theory that the virus of the bee-sting is an infallible remedy for acute rheumatism has received most unquestionable confirmation from the practices of the country people in Malta. Bees are said to be plentiful on the island, and the virtue of the sting as a cure for rheumatism has been long established. It is, in fact, said to have been a common practice for generations past to resort to this remedy in all severe cases, the results being most favorable."

If the foregoing statement proves to be true, and the same virtue dwells in the virus of the sting of the surprisingly active bee of our country, will not some of our brethren, who dwell in the rural districts, give it a practical test and supply the cities with the article?

Very truly yours,

JAMES WOOD, M. D.

BROOKLYN, N. Y.

—*Medical News of Philadelphia, Sept. 9th,* 1893.

